# Supplementary material for: SMARCB1 (INI-1)-Deficient Sinonasal Carcinoma: A Systematic Review and Pooled Analysis of Treatment Outcomes
Source: Cancers (Basel). 2022 Jul 5;14(13):3285. doi: 10.3390/cancers14133285 (PMC9265388; doi:10.3390/cancers14133285)
Supplement: Supplementary file 1 [file cancers-14-03285-s001.zip › cancers-1783227-supplementary.pdf]

cancers

MDPI

*Article*

# SMARCB1 (INI-1)-Deficient Sinonasal Carcinoma: A Systematic Review and Pooled Analysis of Treatment Outcomes

**Victor Ho-Fun Lee, Raymond King-Yin Tsang, Anthony Wing Ip Lo, Sum-Yin Chan, Joseph Chun-Kit Chung, Chi-Chung Tong, To-Wai Leung and Dora Lai-Wan Kwong**

## Supplementary Methods

*Literature search strategy and criteria*

(((((((((((SMARCB1[title/abstract]) OR SMARCB-1[title/abstract]) OR INI1[title/abstract]) OR INI-1[title/abstract]) OR INI?[title/abstract]) OR INI*[title/abstract]) AND sinonasal[title/abstract]) OR paranasal[title/abstract]) OR paranasal sinuses[title/abstract]) OR nasal[title/abstract]))) AND ("0001/01/01"[Date - Publication] : "2022/03/31"[Date - Publication])

*Publications excluded from the systematic review*

1. Agaimy, A. The expanding family of SMARCB1(INI1)-deficient neoplasia: implications of phenotypic, biological, and molecular heterogeneity. *Adv. Anat. Pathol.* **2014**, *21*, 394–410.
**Reason:** It is a general overview article which describes SMARCB1-deficient sinonasal carcinoma as a new disease entity of sinonasal malignancy with a different genetic and molecular characteristics, but without any individual patient data and survival outcomes of SMARCB1-deficient sinonasal carcinoma.

2. Agaimy, A. Poorly differentiated sinonasal tract malignancies: A review focusing on recently described entities. *Cesk. Patol.* **2016**; *52*, 146–153.
**Reason:** It is a general overview article which describes SMARCB1-deficient sinonasal carcinoma as a new disease entity of sinonasal malignancy with a different genetic and molecular characteristics, but without any individual patient data and survival outcomes of SMARCB1-deficient sinonasal carcinoma.

3. Bishop, J.A. Newly described tumor entities in sinonasal tract pathology. *Head Neck Pathol.* **2016**, *10*, 23–31.
**Reason:** It is a general overview article which describes SMARCB1-deficient sinonasal carcinoma as a new disease entity of sinonasal malignancy with a different genetic and molecular characteristics, but without any individual patient data and survival outcomes of SMARCB1-deficient sinonasal carcinoma.

4. Bishop, J.A. Recently described neoplasms of the sinonasal tract. *Semin. Diagn. Pathol.* **2016**, *33*, 62–70.
**Reason:** It is a general overview article which describes SMARCB1-deficient sinonasal carcinoma as a new disease entity of sinonasal malignancy with a different genetic and molecular characteristics, but without any individual patient data and survival outcomes of SMARCB1-deficient sinonasal carcinoma.

5. Barresi, V.; Branca, G.; Raso, A.; Mascelli, S.; Caffo, M.; Tuccari, G. Atypical teratoid rhabdoid tumour involving the nasal cavities and anterior skull base. *Neuropathol.* **2016**, *36*, 283–289.
**Reason:** It is a general overview article of atypical teratoid rhabdoid tumour in the nasal cavities and anterior skull base, without any individual patient data and survival outcomes of SMARCB1-deficient sinonasal carcinoma.

6. Agaimy, A.; Weichert, W. SMARCA4-deficient sinonasal carcinoma. *Head and Neck Pathol.* **2017**, *11*, 541–545.
**Reason:** It is a general overview article of SMARCB4-deficient sinonasal carcinoma, without any individual patient data and survival outcomes of SMARCB1-deficient sinonasal carcinoma.

7. Stelow, E.B.; Bishop. J.A. Update from the 4th Edition of the World Health Organization Classification of head and neck tumours: tumors of the nasal cavity, paranasal sinuses and skull base. *Head and Neck Pathol.* **2017**, *11*, 3–15.
**Reason:** It is a general overview article which describes SMARCB1-deficient sinonasal carcinoma as a new disease entity of sinonasal malignancy, without any individual patient data and survival outcomes of SMARCB1-deficient sinonasal carcinoma.

8. Schaefer, I.M.; Agaimy, A.; Fletcher, C.D.; Hornick, J.L. Claudin-4 expression distinguishes SWI/SNF complex-deficient undifferentiated carcinomas from sarcomas. *Mod. Pathol.* **2017**, *30*, 539–548.
**Reason:** It is a review article which described claudin-4 expression in SWI/SNF complex-deficient (SMARCB1-deficient) undifferentiated carcinoma, without any individual patient data and survival outcomes of SMARCB1-deficient sinonasal carcinoma.

9. Pawel, B.R. SMARCB1-deficient tumors of childhood: a practical guide. *Pediatr. Dev. Pathol.* **2018**, *21*, 6–28.
**Reason:** It is a general overview article of SMARCB1-deficient tumour of childhood, without any individual patient data and survival outcomes of SMARCB1-deficient sinonasal carcinoma.

10. Thompson, L.D.R.; Franchi, A. New tumor entities in the 4th edition of the World Health Organization classification of head and neck tumors: Nasal cavity, paranasal sinuses and skull base. *Virchows Arch.* **2018**, *472*, 315–330.
**Reason:** It is a general overview article which describes SMARCB1-deficient sinonasal carcinoma as a new disease entity of sinonasal malignancy, without any individual patient data and survival outcomes of SMARCB1-deficient sinonasal carcinoma.

11. Laco, J.; Kovarikova, H.; Chmelarova, M.; Vosmikova, H.; Sieglova, K.; Bubancova, I.; Dundr, P.; Nemejcova, K.; Michalek, J, Celakovsky, P.; et al. Analysis of DNA methylation and microRNA expression in NUT (nuclear protein in testis) midline carcinoma of the sinonasal tract: a clinicopathological, immunohistochemical and molecular genetic study. *Neoplasma* **2018**, *65*, 113–123.
**Reason:** It is a journal article which analysed DNA methylation and microRNA expression in NUT midline carcinoma of the sinonasal tract, without any individual patient data and survival outcomes of SMARCB1-deficient sinonasal carcinoma.

12. Agaimy, A.; Weichert, W.; Haller, F.; Hartmann, A. Diagnostic and predictive molecular pathology of head and neck neoplasms. *Pathologe* **2018**, *39*, 42–47.
**Reason:** It is a review article without any individual patient data and survival outcomes of SMARCB1-deficient sinonasal carcinoma. Besides it is not written in English but in German.

13. Agaimy, A.; Haller, F.; Hartmann, A. Sinonasal tumors: News from the WHO with special reference to mesenchymal entities. *Pathologe* **2018**, *39*, 18–26.
**Reason:** It is a review article without any individual patient data and survival outcomes of SMARCB1-deficient sinonasal carcinoma. Besides it is not written in English but in German.

14. McCuiston, A.; Bishop, J.A. Usefulness of NKX2.2 Immunohistochemistry for Distinguishing Ewing Sarcoma from Other Sinonasal Small Round Blue Cell Tumors. *Head Neck Pathol.* **2018**, *12*, 89–94.

**Reason:** It is a journal article which described the use of NKX2.2 immunohistochemistry to distinguish Ewing sarcoma from other sinonasal malignant without any individual patient data and survival outcomes of SMARCB1-deficient sinonasal carcinoma.

15. Rooper, L.M.; Bishop, J.A.; Westra, W.H. INSM1 is a Sensitive and Specific Marker of Neuroendocrine Differentiation in Head and Neck Tumors. *Am. J. Surg. Pathol.* **2018**, *42*, 665–671.
**Reason:** It is a journal article without any individual patient data and survival outcomes of SMARCB1-deficient sinonasal carcinoma.

16. Schaefer, I.M.; Hornick, J.L. Diagnostic Immunohistochemistry for Soft Tissue and Bone Tumors: An Update. *Adv. Anat. Pathol.* **2018**, *25*, 400–412.
**Reason:** It is a review article without any individual patient data and survival outcomes of SMARCB1-deficient sinonasal carcinoma.

17. Silveira, H.A.; Almeida, L.Y.; Nonaka, C.F.W.; Alves, P.M.; Ribeiro-Silva, A.; León, J.E. Myoepithelial carcinoma with rhabdoid features in the maxillary sinus: Immunohistochemical and in situ hybridization analysis of a rare case. *Oral Oncol.* **2019**, *93*, 116–119.
**Reason:** It is a journal article which described myoepithelial carcinoma in the maxillary sinus, without any individual patient data and survival outcomes of SMARCB1-deficient sinonasal carcinoma.

18. Guilmette, J.; Sadow, P.M. High-grade sinonasal carcinoma: classification through molecular profiling. *Arch. Pathol. Lab. Med.* **2019**, *143*, 1416–1419.
**Reason:** It is a review article only without any individual patient data and survival outcomes of SMARCB1-deficient sinonasal carcinoma.

19. Tang, S.J.; Zhai, C.W.; Yuan, C.C.; Zhang, J.H.; Want, S.Y. SMARCB1 (INI1)-deficient sinonasal carcinoma: a clinicopathological analysis of six cases. *Zhonghua Bing Li Xue Za Zhi.* **2020**, *49*, 47–51.
**Reason:** It is not written in English but in Chinese.

20. Neves-Silva, R.; Almeida, L.Y.; Silveira, H.A.; Colturato, C.B.N.; Duarte, A.; Ferrisse, T.M.; Silva, E.V.; Vanzolin, B.F.; Bufalino, A.; Ribeiro-Silva, A.; et al. SMARCB1 (INI-1) and NUT immunoexpression in a large series of head and neck carcinomas in a Brazilian reference center. *Head Neck.* **2020**, *42*, 374–384.
**Reason:** It is a journal article which described SMARCB1 (INI-1) tumours outside the sinonasal region or paranasal sinuses.

21. Agaimy, A.; Franchi, A.; Lund ,V.J.; Skálová, A.; Bishop, J.A; Triantafyllou, A.; Andreasen, S.; Gnepp, D.R.; Hellquist, H.; Thompson, L.D.R.; et al. Sinonasal undifferentiated carcinoma (SNUC): from an entity to morphologic pattern and back again – a historical perspective. *Adv. Anat. Pathol.* **2020**, *27*, 51–60.
**Reason:** It is a review article without any individual patient data and survival outcomes of SMARCB1-deficient sinonasal carcinoma.

22. Agaimy, A.; Jain, D.; Uddin, N.; Rooper, L.M.; Bishop, J.A. SMARCA4-deficient sinonasal carcinoma: a series of 10 cases expanding the genetic spectrum of SWI/SNF-driven sinonasal malignancies. *Am. J. Surg. Pathol.* **2020**, *44*, 703–710.
**Reason:** It is an article on SMARCB4-deficient sinonasal carcinoma, without any individual patient data and survival outcomes of SMARCB1-deficient sinonasal carcinoma.

23. Chen, Z.W.; Fang, S.G.; Ren, J.Q.; Qian, L.J.; Shi, H.Q. Clinical and pathological analysis of 2 cases of SMARCB1(INI-1)-deficient sinonasal carcinoma. *Zhonghua Er Bi Yan Hou Tou Jing Wai Ke Za Zhi.* **2020**, *55*, 400–402.
**Reason:** It is not written in English but in Chinese.

24. Kezlarian, B.E.; Lin, O.; Dogan, S. SMARCB1-deficient carcinomas of the head and neck region: a cytopathologic characterization. *J. Am. Soc. Cytopathol.* **2020**, *9*, 494–501.
**Reason:** It is a review article without any individual patient data and survival outcomes of SMARCB1-deficient sinonasal carcinoma.

25. Heft Neal, M.E.; Birkeland, A.C.; Bhangale, A.D.; Zhai, J.; Kulkarni, A.; Foltin, S.K.; Jewell, B.M.; Ludwig, M.L.; Pinatti, L.; Jiang, H.; et al. Genetic analysis of sinonasal undifferentiated carcinoma discovers recurrent SWI/SNF alterations and a novel PGAP3-SRPK1 fusion gene. *BMC Cancer.* **2021**, *21*, 636.
**Reason:** It is a review article without any individual patient data and survival outcomes of SMARCB1-deficient sinonasal carcinoma.

26. Glöss, S.; Jurmeister, P.; Thieme, A.; Schmid, S.; Cai, W.Y.; Serrette, R.N.; Perner, S.; Ribbat-Idel, J.; Pagenstecher, A.; Bläker, H.; et al. IDH2 R172 Mutations Across Poorly Differentiated Sinonasal Tract Malignancies: Forty Molecularly Homogenous and Histologically Variable Cases With Favorable Outcome. *Am. J. Surg. Pathol.* **2021**, *45*, 1190–1204.
**Reason:** It is a review article without any individual patient data and survival outcomes of SMARCB1-deficient sinonasal carcinoma.

27. Wang, J.Y.; Bai, Y.P.; Xing, L.; Piao, Y.S.; He, X.J.; Yue, C.L.; Zhao, X.L.; Liu, H.G. Clinicopathological characteristics of SMARCB1(INI1)-deficient sinonasal carcinoma. *Zhonghua Bing Li Xue Za Zhi.* **2021**, *50*, 1240–1245.
**Reason:** It is not written in English but in Chinese.

28. Liu, Z.; Seshadri, M.; Gupta, V.; Papanicolau-Sengos, A.; Merzianu, M. INI1-deficient thyroid caricnoma is an aggressive disease with epithelioid and rhabdoid phenotype. A case report, survey of INI1 expression in thyroid lesions and literature review. *Head Neck Pathol.* **2021**, *15*, 1246–1252.
**Reason:** It is a journal article on INI1-deficient thyroid cancer but not on SMARCB1-deficient sinonasal carcinoma.

29. Agaimy, A. Moving from "single gene" concept to "functionally homologous multigene complex": The SWI/SNF paradigm. *Semin. Diagn. Pathol.* **2021**, *38*, 165–166.
**Reason:** It is a review article without any individual patient data and survival outcomes of SMARCB1-deficient sinonasal carcinoma.

30. Agaimy, A.; Bishop, J.A. SWI/SNF-deficient head and neck neoplasms: An overview. *Semin. Diagn. Pathol.* **2021**; 38, 175–182.
**Reason:** It is a review article without any individual patient data and survival outcomes of SMARCB1-deficient sinonasal carcinoma.

31. Lopez, D.C.; Wadley, A.E; London, N.R. Jr. Emerging concepts in sinonasal tumor research. *Curr. Opin. Otolaryngol. Head Neck Surg.* **2022**, *30*, 33–39.
**Reason:** It is a review article without any individual patient data and survival outcomes of SMARCB1-deficient sinonasal carcinoma.

32. Sakthivel, P.; Raveendran, S.; Rajeshwari, M. Sinonasal small round blue cell tumors: What the clinician must know! *Oral Oncol.* **2022**, *126*, 105735.
**Reason:** It is a review article without any individual patient data and survival outcomes of SMARCB1-deficient sinonasal carcinoma.

33. Gonçalves, J.M.; Scarini, J.F.; Gondak, R.; Altemani, A.; Mariano, F.V. Oral involvement of sinonasal undifferentiated carcinoma: A case report and immunohistochemical study of a challenging case. *Oral Oncol.* **2022**, *126*, 105779.
**Reason:** It is a case report describing a SMARCB1-positive sinonasal carcinoma.

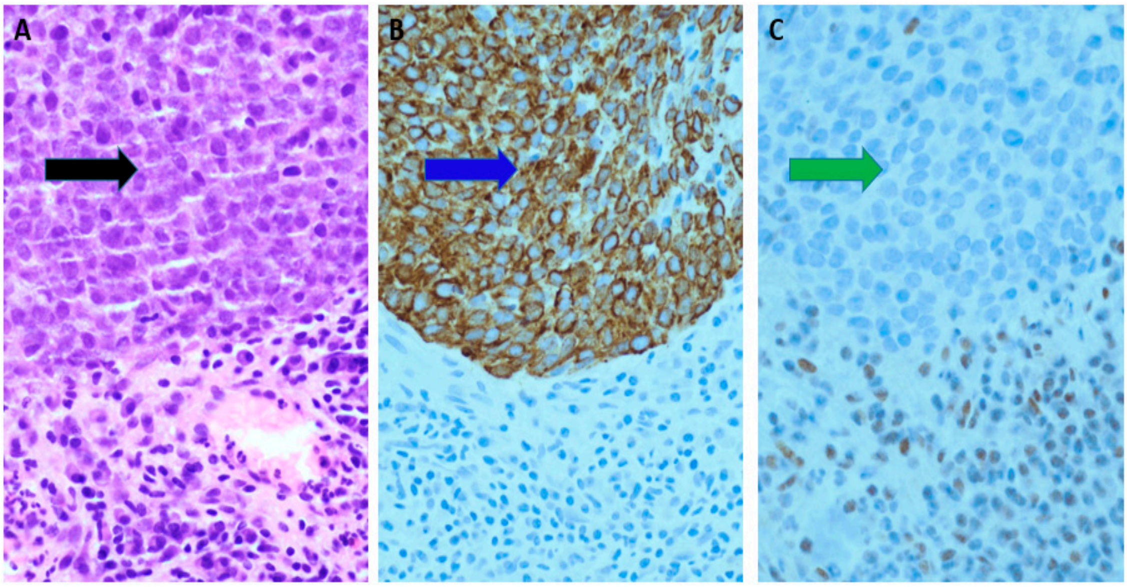


**Figure S1.** The tumor of our patient (Case number 124 in the Table S2) shows (**A**) a predominant basaloid cell pattern (haematoxylin and eosin x 400, black arrow)**.** (**B**) These tumor cells are strongly immunoreactive to pan-cytokeratins (immunohistochemical staining for cytokeratins MNF116, blx400, blue arrow). (**C**) Loss of expression of SMACRB1 is demonstrated, contrasting with retained signals in the stromal inflammatory cells (immunohistochemical staining for INI-1, x400, green arrow).

FOUNDATION**ONE**

| Patient Name | Report Date | Tumor Type |
|---|---|---|
| | 20 July 2018 | Nasopharynx and paranasal sinuses undifferentiated carcinoma |

| | | | | | |
|---|---|---|---|---|---|
| Date of Birth | | Medical Facility | Queen Mary Hospital Hong Kong | | |
| Sex | | Ordering Physician | Lee, Victor | Specimen Received | 09 July 2018 |
| FMI Case # | | Additional Recipient | | Specimen Site | Nasal Cavity |
| Medical Record # | | Medical Facility ID # | | Date of Collection | 28 May 2018 |
| Specimen ID | | Pathologist | | Specimen Type | Slide |

## ABOUT THE TEST:

FoundationOne™ is a next-generation sequencing (NGS) based assay that identifies genomic alterations within hundreds of cancer-related genes.

## PATIENT RESULTS

3 genomic findings

0 therapies associated with potential clinical benefit

0 therapies associated with lack of response

10 clinical trials

## TUMOR TYPE: NASOPHARYNX AND PARANASAL SINUSES UNDIFFERENTIATED CARCINOMA

**Genomic Alteration Identified†**

*SMARCB1* loss

**Additional Findings†**

*Microsatellite status* MS-Stable

*Tumor Mutational Burden* TMB-Low; 1 Muts/Mb

† For a complete list of the genes assayed and performance specifications, please refer to the Appendix

## THERAPEUTIC IMPLICATIONS

| Genomic Findings Detected | FDA-Approved Therapies (in patient's tumor type) | FDA-Approved Therapies (in another tumor type) | Potential Clinical Trials |
|---|---|---|---|
| ***SMARCB1*** loss | None | None | Yes, see clinical trials section |
| ***Microsatellite status*** MS-Stable | None | None | None |
| ***Tumor Mutational Burden*** TMB-Low; 1 Muts/Mb | None | None | None |

Note: Genomic alterations detected may be associated with activity of certain FDA-approved drugs; however, the agents listed in this report may have little or no evidence in the patient's tumor type. Neither the therapeutic agents nor the trials identified are ranked in order of potential or predicted efficacy for this patient, nor are they ranked in order of level of evidence for this patient's tumor type.

**For more comprehensive information please log on to the Interactive Cancer Explorer™**
**To set up your Interactive Cancer Explorer account, contact your sales representative or call 1-888-988-3639.**

Electronically Signed by Jo-Anne Vergilio, M.D. | Jeffrey S. Ross, M.D., Medical Director | 20 July 2018
Foundation Medicine, Inc. / 1-888-988-3639

Sample Preparation: 7010 Kit Creek Road, Morrisville, NC 27560 / CLIA: 34D2044309
Sample Analysis: 150 Second St., 1st Floor, Cambridge, MA 02141 / CLIA: 22D2027531

page 1 of 18

FOUNDATION**ONE**®

Patient Name

Report Date
**20 July 2018**

Tumor Type
**Nasopharynx and paranasal sinuses undifferentiated carcinoma**

## GENOMIC ALTERATIONS

| GENE ALTERATION | INTERPRETATION |
|---|---|
| ***SMARCB1*** loss | **Gene and Alteration:** The SMARCB1 gene encodes the SNF5 protein, also known as INI1, one of three core subunits of the SWI/SNF family of chromatin remodeling complexes[1]. SNF5, as a member of the remodeling complex, plays a key role in cell cycle control and can act as a tumor suppressor[2]. Alterations in SMARCB1 that remove or disrupt the DNA binding domain (amino acids 106-183) or protein-protein interaction domains (amino acids 186-318)[3,4,5], as observed here, are predicted to be inactivating.<br><br>**Frequency and Prognosis:** In a genomic analysis of 56 nasopharyngeal carcinoma cases, alterations of SMARCB1 was not detected in any of the samples[6]. Loss of SMARCB1 has been reported in all three sinonasal basaloid carcinomas analyzed in one study[7]. Additionally, the loss of SMARCB1 protein expression was reported in 6% of 142 sinonasal carcinomas in another study, with genomic loss of SMARCB1 identified in 75% (6/8) of patients in that subset[8]. These studies suggest that SMARCB1 loss may indicate a sinonasal carcinoma characterized by poor differentiation and aggressive clinicopathologic features.<br><br>**Potential Treatment Strategies:** There are no therapies that directly target SMARCB1 loss or inactivating mutations. Preclinical evidence suggests that tumors with loss of SMARCB1 may be sensitive to a variety of targeted therapies, including EZH2 inhibitors[9,10], inhibitors of the hedgehog pathway[11], CDK4/6 inhibitors[12,13], proteasome inhibitors[14], inhibitors of fibroblast growth receptors (FGFRs)[15], and Aurora kinase inhibitors[16]. Several of these agents are being tested in clinical trials. A clinical trial of the Aurora kinase A inhibitor alisertib for pediatric patients with atypical teratoid rhabdoid tumors, which are characterized by SMARCB1 loss or inactivation, reported disease stabilization or regression in 100% (4/4) of patients, and 2 patients achieved disease regression lasting more than 1 year[17]. EZH2 inhibitor tazemetostat (EPZ-6438) has shown efficacy, including several complete responses, in adult and pediatric patients with INI1-negative tumors in early phase trials (Gounder et al., 2017; ASCO Abstract 11058 , Chi et al., 2017; AACR-NCI-EORTC Abstract 175, taliano et al., 2015; ECC Abstract 302), with one study of INI1-negative epithelioid sarcoma reporting 4 partial responses and 18 stable disease versus 7 progressive disease, with median progression-free survival of 5.7 months (Gounder et al., 2017; ASCO Abstract 11058). Clinical data suggest that CDK4/6 inhibitors, such as ribociclib, abemaciclib, and palbociclib, have efficacy for the treatment of solid tumors[18,19,20,21,22]. |
| ***Microsatellite status*** MS-Stable | **Gene and Alteration:** Microsatellite instability (MSI) is a condition of genetic hypermutability that generates excessive amounts of short insertion/deletion mutations in the genome; it generally occurs at microsatellite DNA sequences and is caused by a deficiency in DNA mismatch repair (MMR) in the tumor[23]. Defective MMR and consequent MSI occur as a result of genetic or epigenetic inactivation of one of the MMR pathway proteins, primarily MLH1, MSH2, MSH6, or PMS2[23,24,25]. The tumor seen here is microsatellite-stable (MSS), equivalent to the clinical definition of an MSS tumor: one with mutations in none of the tested microsatellite markers[26,27,28]. MSS status indicates MMR proficiency and typically correlates with intact expression of all MMR family proteins[23,25,27,28]. |

**For more comprehensive information please log on to the Interactive Cancer Explorer™**
**To set up your Interactive Cancer Explorer account, contact your sales representative or call 1-888-988-3639.**

**Electronically Signed by Jo-Anne Vergilio, M.D. | Jeffrey S. Ross, M.D., Medical Director | 20 July 2018**
Foundation Medicine, Inc. / 1-888-988-3639

**Sample Preparation:** 7010 Kit Creek Road, Morrisville, NC 27560 / CLIA: 34D2044309
**Sample Analysis:** 150 Second St., 1st Floor, Cambridge, MA 02141 / CLIA: 22D2027531

page 2 of 18

**Figure S2.** Comprehensive genomic profiling with FoundationOne® of the tumour in our patient (Case number 124 in the Table S2) revealing a complete genomic loss of SMARCB1.

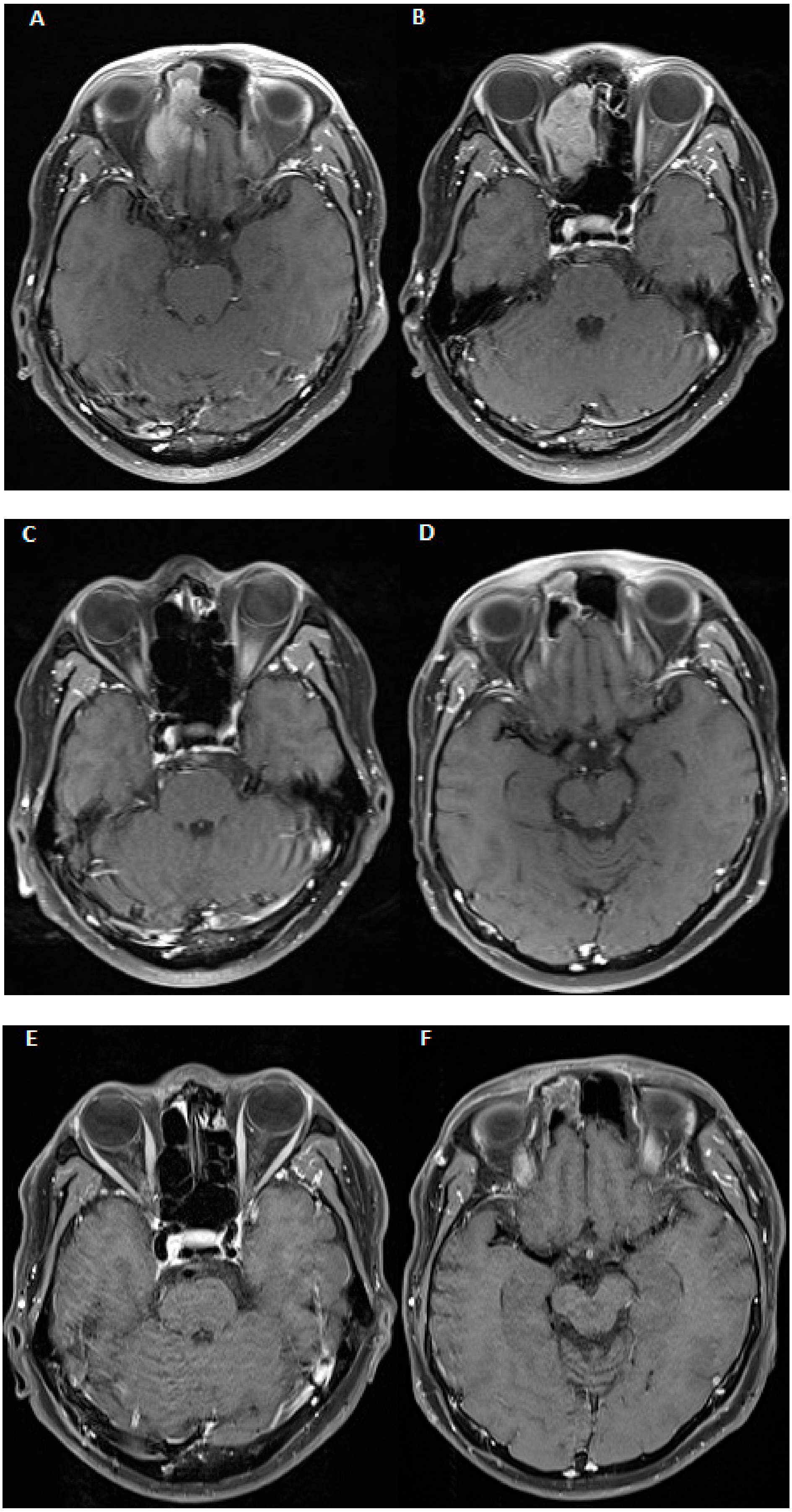
A
B
C
D
E
F

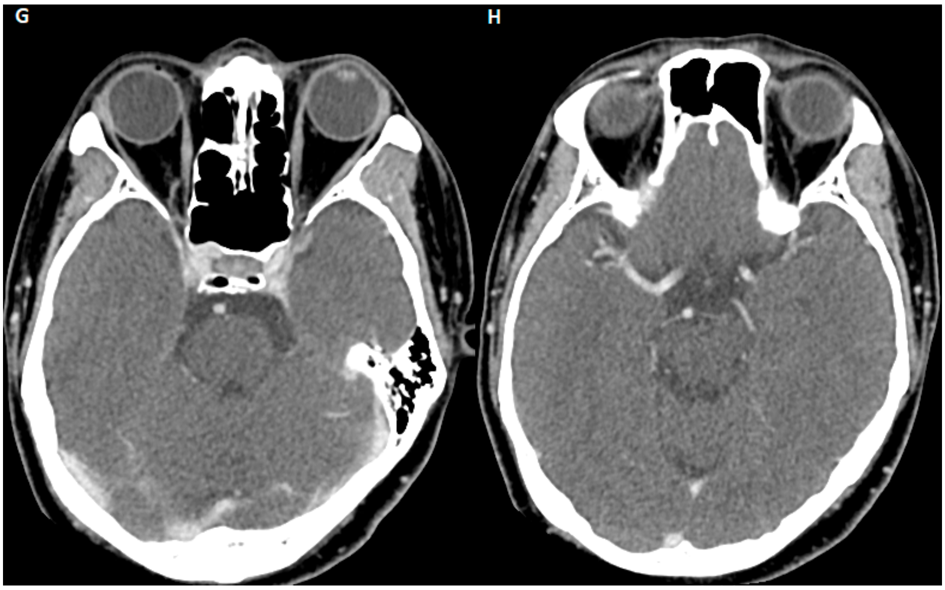


**Figure S3.** Radiological images of our patient (Case number 124 in the Table S2) treated at our institution. (**A**) and (**B**) Magnetic resonance imaging of the paranasal sinuses showing a T1-weighted fat-saturated contrast-enhancing soft tissue mass in the right ethmoid and frontal sinuses invading the lamina papyracea and compressing the right medial rectus muscle at the time of histological diagnosis of SMARCB1-deficient sinonasal carcinoma. (**C**) and (**D**) Magnetic resonance imaging of the paranasal sinus at 12 weeks after completion of induction chemotherapy with docetaxel, cisplatin and 5-fluorouracil (TPF) and radical concurrent chemoradiation with photons of 60Gy in 30 fractions and 2 cycles of 3-weekly cisplatin demonstrating a complete radiological response. (**E**) and (**F**) Magnetic resonance imaging at 6 months after induction chemotherapy and concurrent chemoradiation demonstrating persistent complete response. (**G**) and (**H**) Contrast-enhanced computed tomography of the paranasal sinus at 2.5 years after induction chemotherapy and concurrent chemoradiation demonstrating persistent complete response.

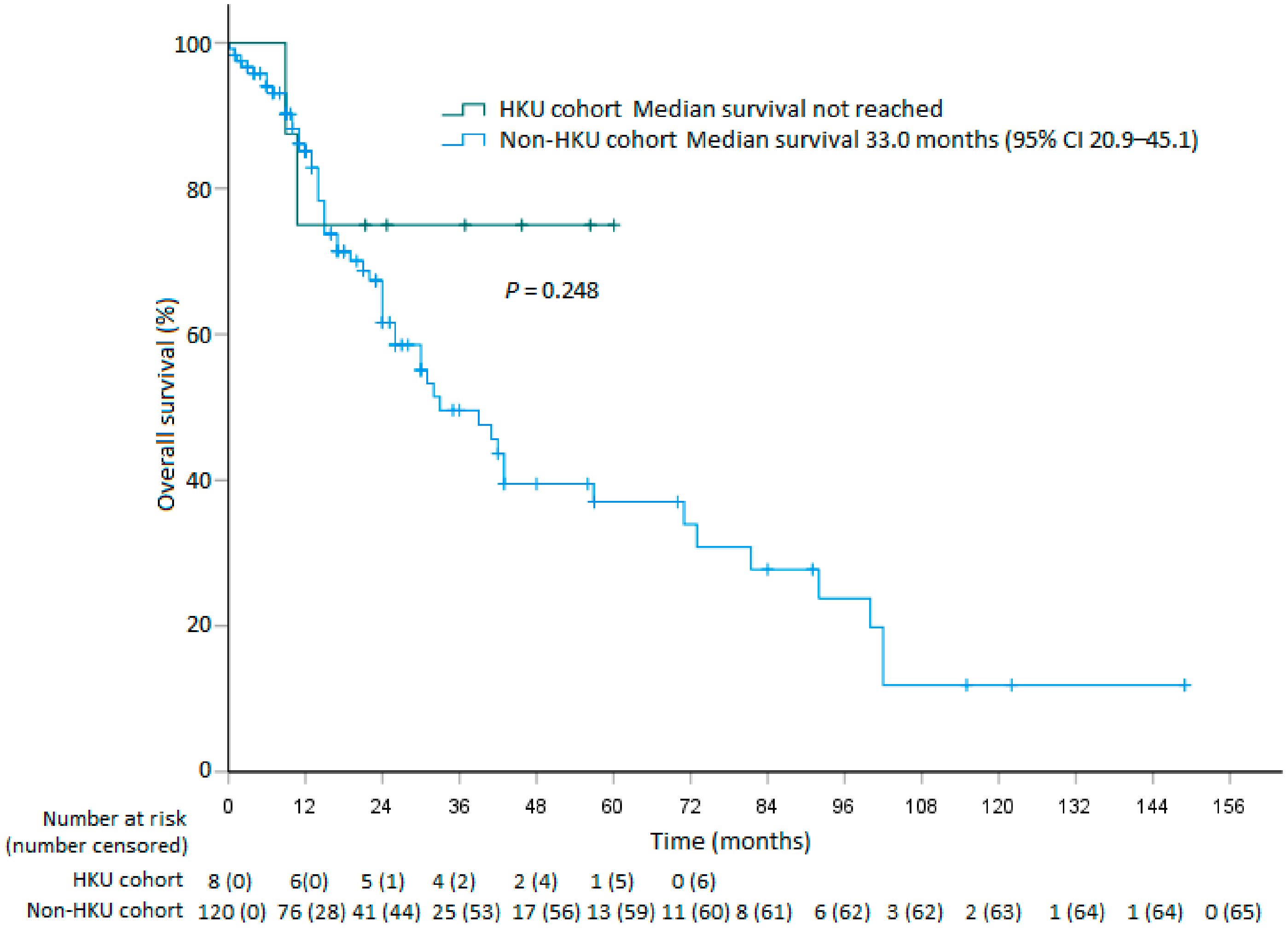


**Figure S4.** Kaplan-Meier estimates of overall survival of patients with SMARCB1-deficient sinonasal carcinoma identified from systematic review and those treated at our institution.

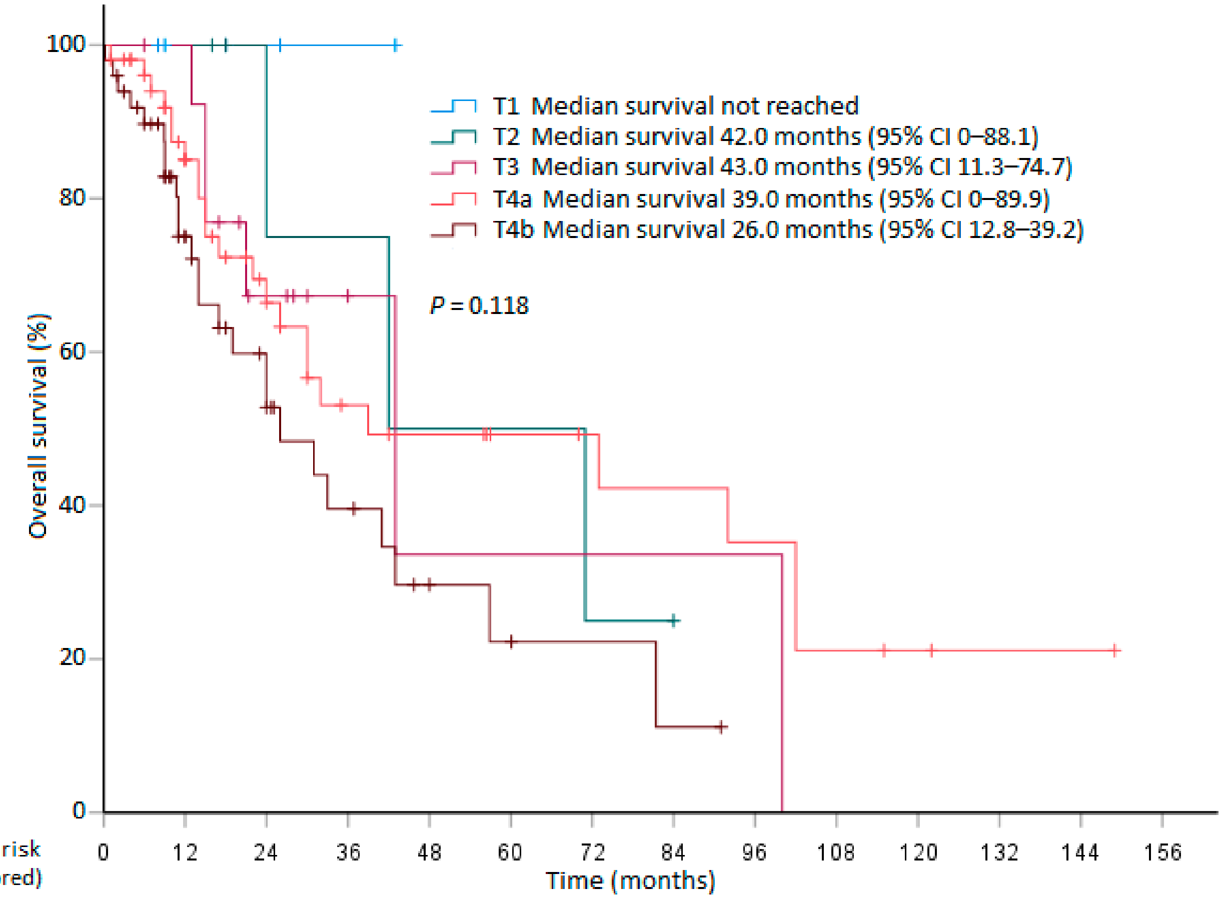


| Number at risk (number censored) | 0 | 12 | 24 | 36 | 48 | 60 | 72 | 84 | 96 | 108 | 120 | 132 | 144 | 156 |
|---|---|---|---|---|---|---|---|---|---|---|---|---|---|---|
| T1 | 5 (0) | 3 (2) | 2 (3) | 1 (4) | 0 (5) | | | | | | | | | |
| T2 | 6 (0) | 6 (0) | 3 (2) | 3 (2) | 2 (2) | 2 (2) | 1 (2) | 0 (3) | | | | | | |
| T3 | 14 (0) | 13 (1) | 6 (4) | 2 (8) | 1 (8) | 1 (8) | 1 (8) | 1 (8) | 1 (8) | 0 (8) | | | | |
| T4a | 53 (0) | 34 (12) | 21 (18) | 14 (21) | 12 (22) | 8 (26) | 7 (27) | 6 (27) | 5 (27) | 3 (27) | 2 (28) | 1 (29) | 1 (29) | 0 (30) |
| T4b | 50 (0) | 26 (13) | 14 (18) | 9 (20) | 4 (23) | 3 (23) | 2 (24) | 1 (24) | 0 (25) | | | | | |

**Figure S5.** Kaplan-Meier estimates of overall survival of patients with SMARCB1-deficient sinonasal carcinoma stratified by T-categories.

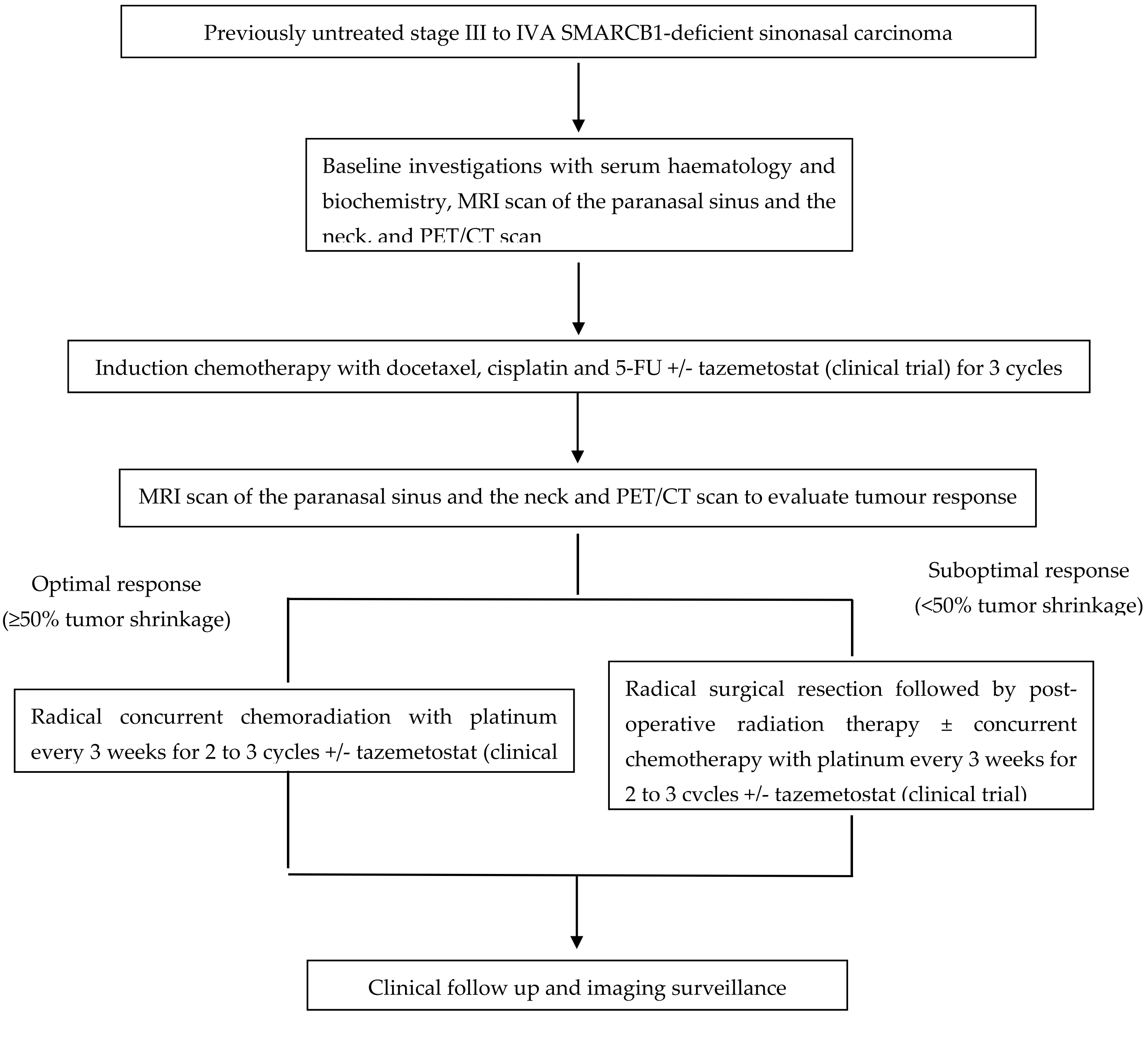


**Figure S6.** Our proposed treatment strategy for previously untreated stage III to IVA SMARCB1-deficient sinonasal carcinoma.

**Table S1.** T and N categories for tumors of the nasal cavity and paranasal sinuses according to the 8th edition of American Joint Committee on Cancer/Union for International Cancer Control TNM classification system.

| T category | Maxillary sinus | Nasal cavity and ethmoid sinus | N category | Lymph node metastasis |
|---|---|---|---|---|
| Tis | Carcinoma in-situ | Carcinoma in-situ | N0 | No regional lymph node metastasis |
| T1 | Tumor limited to the mucosal lining | Tumor limited to the mucosal lining | N1 | Metastasis in a single ipsilateral lymph node, 3cm or less in greatest dimension |
| T2 | Bone erosion or destruction limited to the hard palate and middle meatus | Tumor at 2 subsites or adjacent nasoethmoid sites | N2a | Metastasis in a single ipsilateral lymph node 3–6cm |
| T3 | Bone erosion or destruction of the posterior bone of maxillary sinus, floor and medial bone of orbit. Tumor growth into the pterygoid fossa or ethmoid sinus | Bone erosion of lamina papyracea or floor of orbit, maxillary sinus, palate and cribriform plate | N2b | Metastases in multiple ipsilateral lymph nodes <6cm |
| T4a | Tumor growth into the anterior orbit, pterygoid plates, infratemporal fossa, cribriform plate, frontal sinus, sphenoid sinus, or skin of cheek | Tumor growth into the anterior orbit, anterior cranial fossa, pterygoid fossa, pterygoid plates, frontal sinus, sphenoid sinus, or skin of nose or cheek | N2c | Metastases in bilateral or contralateral lymph nodes <6cm |
| T4b | Tumor growth into the orbital apex, dura mater, brain middle cranial fossa, cranial nerves other than V2, nasopharynx, or clivus | | N3 | Metastasis in a lymph node >6cm |

**Table S2.** Details of all 128 patients with SMARCB1-deficient sinonasal carcinoma.

| Case number | Author* | Year | Age at diagnosis | Sex (0=male; 1=female) | T-category | N-category | M-category | Tumour extent | Treatment | Induction treatment (0=no; 1=yes) | Adjuvant treatment (0=no; 1=yes) | Preoperative treatment, operation and postoperative treatment (0=no; 1=yes) | Multimodality treatment (0=no; 1=yes) | Relapse (0=no; 1=yes) | Local relapse (0=no; 1=yes) | Regional relapse (0=no; 1=yes_ | Distant relapse (0=no; 1=yes) | Survival status (0=alive; 1=dead) | Duration of survival (months) | Disease status at last follow up (0=no evidence of disease; 1=persistent disease; 2=died of disease; 3=died of other causes) | Predominant histological features (if available) |
|---|---|---|---|---|---|---|---|---|---|---|---|---|---|---|---|---|---|---|---|---|---|
| 1 | Agaimy et al,[1] | 2014 | 52 | 0 | 4a | 1 | 0 | Nasal cavity, left frontal and ethmoidal sinuses | Radical resection of the sinonasal lesion and neck dissection followed by postoperative concurrent chemoradiation | 0 | 1 | 0 | 1 | 1 | 0 | 1 | 0 | 1 | 102.0 | 2 | - |
| 2 | Agaimy et al,[1] | 2014 | 28 | 1 | 2 | 0 | 0 | Nasal cavity, right frontal sinus and anterior ethmoid sinus | Multiple local resections | 0 | 0 | 0 | 0 | 0 | 0 | 0 | 0 | 0 | 84.0 | 0 | - |
| 3 | Agaimy et al,[1] | 2014 | 35 | 1 | 4a | 0 | 0 | Right anterior ethmoidal sinus with invasion into the frontal sinuses and | Radical resection of the tumor with enucleation of the ocular bulb and resection of | 0 | 1 | 0 | 1 | 1 | 0 | 0 | 1 | 0 | 70.0 | 1 | - |

| | | | | | | | | | | | | | | | | | | | | | |
|---|---|---|---|---|---|---|---|---|---|---|---|---|---|---|---|---|---|---|---|---|---|
| | | | | | | | | the right medial orbital wall | the bony wall of the ethmoid sinus followed by postoperative chemoradiation | | | | | | | | | | | | |
| 4 | Bishop et al,[2] | 2014 | 54 | 1 | 4a | 0 | 0 | Nasal cavity, ethmoidal and maxillary sinuses | Radical resection followed by postoperative chemoradiation | 0 | 1 | 0 | 1 | 0 | 1 | 0 | 0 | 1 | 15.0 | 2 | Undifferentiated |
| 5 | Bishop et al,[2] | 2014 | 71 | 1 | 3 | 0 | 0 | Maxillary sinus | Radical resection followed by postoperative radiation therapy | 0 | 1 | 0 | 1 | 1 | 1 | 0 | 0 | 1 | 15.0 | 2 | High-grade non-intestinal adenocarcinoma |
| 6 | Bishop et al,[2] | 2014 | 33 | 0 | 4a | 0 | 0 | Nasal, ethmoidal and frontal sinuses | Radical resection followed by postoperative radiation therapy | 0 | 1 | 0 | 1 | 1 | 1 | 0 | 1 | 1 | 30.0 | 2 | Undifferentiated carcinoma |
| 7 | Bishop et al,[2] | 2014 | 60 | 0 | 4a | 0 | 0 | Ethmoidal sinus | Radical resection followed by postoperative radiation therapy | 0 | 1 | 0 | 1 | 0 | 0 | 0 | 0 | 1 | 10.0 | 0 | Squamous |
| 8 | Bishop et al,[2] | 2014 | 67 | 1 | 4a | 0 | 0 | Ethmoidal sinus | Radical resection followed by chemoradiation | 0 | 1 | 0 | 1 | 1 | 1 | 1 | 0 | 0 | 16.0 | 0 | Undifferentiated |
| 9 | Bishop et al,[2] | 2014 | 46 | 1 | 4a | 0 | 0 | Frontal and ethmoid sinuses | Resection followed by radiation therapy | 0 | 1 | 0 | 1 | 0 | 0 | 0 | 0 | 0 | 57.0 | 0 | Squamous |

| | | | | | | | | | | | | | | | | | | | | | |
|---|---|---|---|---|---|---|---|---|---|---|---|---|---|---|---|---|---|---|---|---|---|
| 10 | Jamshidi et al,[11] | 2014 | 32 | 0 | 4b | 0 | 0 | Sphenoid sinus with compression of bilateral optic nerves | Debulking surgery followed by chemotherapy TPF x 2 cycles and chemoradiation with concurrent weekly cisplatin | 0 | 1 | 0 | 1 | 1 | 1 | 0 | 1 | 1 | 6.0 | 2 | Squamous |
| 11 | Allison et al,[13] | 2016 | 77 | 0 | 4b | 1 | 0 | Posterior ethmoidal sinus invading into skull base and orbit | Radical chemoradiation | 0 | 0 | 0 | 1 | 0 | 1 | 1 | 1 | 1 | 24.0 | 2 | Plasmacytoid/rhabdoid |
| 12 | Zeng et al,[14] | 2016 | 86 | 0 | 3 | 0 | 0 | Left nasal cavity, left maxillary sinus with invasion of maxillary bone | Radical resection | 0 | 0 | 0 | 0 | 0 | 0 | 0 | 0 | 1 | 21.0 | 0 | Basaloid |
| 13 | Shatzkes et al,[15] | 2017 | 77 | 0 | 4b | 0 | 0 | Nasal cavity, ethmoidal, maxillary and sphenoidal sinuses | Radical resection followed by postoperative chemoradiation | 0 | 1 | 0 | 1 | 1 | 1 | 0 | 0 | 1 | 17.0 | 2 | Plasmacytoid/Rhabdoid |
| 14 | Shatzkes et al,[15] | 2017 | 51 | 1 | 4a | 0 | 0 | Sphenoidal and ethmoidal sinuses | Radical resection followed by postoperative chemoradiation | 0 | 1 | 0 | 1 | 1 | 1 | 0 | 1 | 1 | 24.0 | 2 | Undifferentiated carcinoma |
| 15 | Shatzkes et al,[15] | 2017 | 33 | 1 | 4b | 0 | 0 | Nasal cavity and | Radical resection followed by | 0 | 1 | 0 | 1 | 1 | 1 | 0 | 0 | 1 | 33.0 | 2 | Germ cell tumour |

| | | | | | | | | | | | | | | | | | | | | | |
|---|---|---|---|---|---|---|---|---|---|---|---|---|---|---|---|---|---|---|---|---|---|
| | | | | | | | | ethmoidal sinus | chemoradiation | | | | | | | | | | | | |
| 16 | Shatzkes et al,[15] | 2017 | 51 | 0 | 4a | 0 | 0 | Nasal cavity | Surgery followed by chemoradiation | 0 | 1 | 0 | 1 | 0 | 0 | 0 | 0 | 0 | 1.0 | 0 | Adenocarcinoma |
| 17 | Shatzkes et al,[15] | 2017 | 62 | 0 | 4b | 0 | 1 | Nasal cavity and ethmoidal sinus | Radical resection followed by postoperative chemoradiation | 0 | 1 | 0 | 1 | 0 | 0 | 0 | 0 | 0 | 3.0 | 0 | Undifferentiated carcinoma |
| 18 | Shatzkes et al,[15] | 2017 | 54 | 1 | 4b | 0 | 0 | Nasal cavity and ethmoidal sinus | Radical resection followed by postoperative chemoradiation | 0 | 1 | 0 | 1 | 1 | 1 | 0 | 0 | 0 | 6.0 | 1 | Undifferentiated carcinoma |
| 19 | Shatzkes et al,[15] | 2017 | 43 | 0 | 1 | 0 | 0 | Nasal cavity | Radical resection followed by postoperative chemoradiation | 0 | 1 | 0 | 1 | 0 | 0 | 0 | 0 | 0 | 9.0 | 0 | Squamous |
| 20 | Shatzkes et al,[15] | 2017 | 50 | 1 | 4a | 0 | 0 | Nasal cavity | Radical resection followed by postoperative chemoradiation | 0 | 1 | 0 | 1 | 0 | 0 | 0 | 0 | 0 | 9.0 | 0 | Squamous papillary |
| 21 | Shatzkes et al,[15] | 2017 | 35 | 1 | 4b | 0 | 0 | Nasal cavity and ethmoidal sinus | Radical resection followed by postoperative chemoradiation | 0 | 1 | 0 | 1 | 0 | 0 | 0 | 0 | 0 | 10.0 | 0 | Squamoid |
| 22 | Shatzkes et al,[15] | 2017 | 59 | 0 | 4a | 0 | 0 | Sphenoidal and ethmoidal sinuses | Radical resection followed by postoperative | 0 | 1 | 0 | 1 | 0 | 0 | 0 | 0 | 0 | 12.0 | 0 | - |

| | | | | | | | | | | | | | | | | | | | | | |
|---|---|---|---|---|---|---|---|---|---|---|---|---|---|---|---|---|---|---|---|---|---|
| | | | | | | | | | chemoradiation | | | | | | | | | | | | |
| 23 | Shatzkes et al,[15] | 2017 | 72 | 0 | 4a | 0 | 0 | Nasal cavity | Radical resection followed by postoperative chemoradiation | 0 | 1 | 0 | 1 | 0 | 0 | 0 | 0 | 0 | 12.0 | 0 | Undifferentiated carcinoma |
| 24 | Shatzkes et al,[15] | 2017 | 45 | 0 | 4b | 0 | 0 | Nasal cavity and ethmoidal sinus | Radical resection followed by postoperative chemoradiation | 0 | 1 | 0 | 1 | 0 | 0 | 0 | 0 | 0 | 48.0 | 0 | Adenocarcinoma |
| 25 | Shatzkes et al,[15] | 2017 | 75 | 0 | 4b | 0 | 0 | Nasal cavity and ethmoidal sinus | Preoperative chemoradiation followed by surgery and postoperative radiation therapy | 1 | 1 | 1 | 1 | 1 | 1 | 1 | 0 | 0 | 12.0 | UD | Basaloid |
| 26 | Shatzkes et al,[15] | 2017 | 78 | 1 | 1 | 0 | 0 | Nasal cavity | Radical resection | 0 | 0 | 0 | 0 | 0 | 0 | 0 | 0 | 0 | 26.0 | 0 | Plasmacytoid/rhabdoid |
| 27 | Shatzkes et al,[15] | 2017 | 44 | 0 | 4b | 0 | 0 | Nasal cavity and ethmoidal sinus | Radical resection followed by postoperative radiation therapy | 0 | 1 | 0 | 1 | 0 | 0 | 0 | 0 | 0 | 18.0 | 0 | Basaloid |
| 28 | Shatzkes et al,[15] | 2017 | 32 | 0 | 4b | 0 | 0 | Nasal cavity and ethmoidal sinus | Radical resection followed by postoperative radiation therapy | 0 | 1 | 0 | 1 | 1 | 1 | 0 | 0 | 0 | 24.0 | 1 | Undifferentiated carcinoma |
| 29 | Shatzkes et al,[15] | 2017 | 64 | 1 | 4b | 0 | 0 | Frontal and ethmoidal sinuses with invasion into the orbit | Radical resection followed by postoperative chemoradiation | 0 | 1 | 0 | 1 | 1 | 1 | 0 | 0 | 0 | 13.0 | 1 | Undifferentiated carcinoma |

| | | | | | | | | | | | | | | | | | | | | | |
|---|---|---|---|---|---|---|---|---|---|---|---|---|---|---|---|---|---|---|---|---|---|
| 30 | Agaimy et al,[16] | 2017 | 60 | 0 | 4a | 0 | 0 | Ethmoidal sinus | Radical resection followed by postoperative radiation therapy | 0 | 1 | 0 | 1 | 0 | 0 | 0 | 0 | 1 | 10.0 | UD | Basaloid |
| 31 | Agaimy et al,[16] | 2017 | 24 | 1 | 4a | 0 | 0 | Nasal cavity, ethmoidal and sphenoid sinuses | Chemotherapy | 0 | 0 | 0 | 0 | 0 | 0 | 0 | 0 | 1 | 1.0 | 2 | Plasmacytoid/rhabdoid |
| 32 | Agaimy et al,[16] | 2017 | 61 | 0 | 3 | 0 | 0 | Nasal cavity and ethmoidal sinus | Preoperative chemoradiation followed by radical resection | 1 | 0 | 0 | 1 | 1 | 1 | 1 | 1 | 1 | 43.0 | 2 | Basaloid |
| 33 | Agaimy et al,[16] | 2017 | 35 | 1 | 4a | 0 | 0 | Right anterior ethmoidal and bilateral frontal sinuses | Radical resection followed by postoperative chemoradiation | 0 | 1 | 0 | 1 | 1 | 0 | 1 | 1 | 1 | 93.0 | 2 | Basaloid SCC |
| 34 | Agaimy et al,[16] | 2017 | 52 | 0 | 4a | 2 | 0 | Ethmoidal and left frontal sinuses | Radical resection followed by postoperative chemoradiation | 0 | 1 | 0 | 1 | 1 | 0 | 1 | 0 | 1 | 102.0 | 2 | Plasmacytoid/rhabdoid |
| 35 | Agaimy et al,[16] | 2017 | 21 | 0 | 4a | UK | UK | Nasal cavity | Radical resection | 0 | 0 | 0 | 0 | 0 | 0 | 0 | 0 | 1 | 6.0 | 2 | Basaloid |
| 36 | Agaimy et al,[16] | 2017 | 67 | 1 | 3 | 0 | 0 | Right ethmoidal, sphenoidal, and frontal sinuses | Radical resection followed by postoperative chemoradiation | 0 | 1 | 0 | 1 | 1 | 0 | 0 | 1 | 1 | 100.0 | 2 | Basaloid |
| 37 | Agaimy et al,[16] | 2017 | 24 | 0 | 4a | UK | UK | Frontal sinus | Radical resection followed by postoperative | 0 | 1 | 0 | 1 | 1 | 1 | 0 | 1 | 1 | 14.0 | 2 | Basaloid |

| | | | | | | | | | | | | | | | | | | | | |
|---|---|---|---|---|---|---|---|---|---|---|---|---|---|---|---|---|---|---|---|---|
| | | | | | | | | | chemoradiation | | | | | | | | | | | |
| 38 | Agaimy et al,[16] | 2017 | 77 | 0 | 4a | 0 | 0 | Ethmoidal sinus | Radical resection followed by postoperative chemoradiation | 0 | 1 | 0 | 1 | 1 | 0 | 0 | 1 | 1 | 17.0 | 2 | Plasmacytoid/rhabdoid |
| 39 | Agaimy et al,[16] | 2017 | 54 | 1 | 4a | 0 | 0 | Nasal cavity, ethmoidal and maxillary sinuses | Radical resection followed by postoperative chemoradiation | 0 | 1 | 0 | 1 | 1 | 1 | 0 | 0 | 1 | 15.0 | 2 | Basaloid |
| 40 | Agaimy et al,[16] | 2017 | 67 | 0 | 4a | 1 | 0 | Left maxillary, sphenoidal and frontal sinuses | Radical resection followed by postoperative radiation therapy and palliative chemotherapy | 0 | 1 | 0 | 1 | 1 | 1 | 0 | 1 | 1 | 22.0 | 2 | Basaloid |
| 41 | Agaimy et al,[16] | 2017 | 71 | 1 | 3 | 0 | 0 | Maxillary sinus | Radical resection followed by postoperative radiation therapy | 0 | 1 | 0 | 1 | 1 | 1 | 0 | 0 | 1 | 15.0 | 2 | Basaloid, spindled, adenoid |
| 42 | Agaimy et al,[16] | 2017 | 33 | 0 | 4a | 0 | 0 | Nasal cavity, ethmoidal and frontal sinuses | Radical resection followed by postoperative radiation therapy | 0 | 1 | 0 | 1 | 1 | 1 | 0 | 1 | 1 | 30.0 | 2 | Basaloid |
| 43 | Agaimy et al,[16] | 2017 | 86 | 0 | 4b | 0 | 0 | Nasal cavity, maxillary and ethmoid sinuses with | Preoperative radiation therapy followed by radical resection | 1 | 0 | 0 | 1 | 1 | 1 | 1 | 1 | 1 | 9.0 | 2 | Plasmacytoid/rhabdoid |

| | | | | | | | | | | | | | | | | | | |
|---|---|---|---|---|---|---|---|---|---|---|---|---|---|---|---|---|---|---|
| | | | | | | | intracranial extension | | | | | | | | | | | |
| 44 | Agaimy et al,[16] | 2017 | 89 | 0 | 3 | 0 | 0 | Nasal cavity, sphenoidal and ethmoid sinuses | Radical resection followed by postoperative radiation therapy | 0 | 1 | 0 | 1 | 1 | 1 | 0 | 0 | 0 | 6.0 | 2 | Plasmacytoid/rhabdoid |
| 45 | Agaimy et al,[16] | 2017 | 40 | 1 | 4a | 0 | 0 | Left nasal cavity and middle turbinate, ethmoidal and frontal sinuses | Radical chemoradiation | 0 | 0 | 0 | 1 | 1 | 1 | 0 | 0 | 0 | 24.0 | 1 | Plasmacytoid/rhabdoid |
| 46 | Agaimy et al,[16] | 2017 | 38 | 1 | 4a | 0 | 0 | All paranasal sinuses with skull base invasion | Biopsy followed by chemotherapy | 0 | 0 | 0 | 0 | 0 | 0 | 0 | 0 | 0 | 4.0 | 1 | Plasmacytoid/rhabdoid |
| 47 | Agaimy et al,[16] | 2017 | 70 | 0 | 4a | 0 | 0 | Ethmoidal and sphenoidal sinuses | Chemotherapy | 0 | 0 | 0 | 0 | 1 | 1 | 0 | 0 | 0 | 9.0 | 1 | Basaloid |
| 48 | Agaimy et al,[16] | 2017 | 64 | 0 | 1 | UK | UK | Nasal cavity | Radical chemoradiation | 0 | 0 | 0 | 1 | 0 | 0 | 0 | 0 | 0 | 18.0 | 0 | Basaloid |
| 49 | Agaimy et al,[16] | 2017 | 46 | 0 | 1 | 0 | 0 | Frontal sinus | Preoperative chemoradiation, followed by radical resection and postoperative chemotherapy | 1 | 1 | 1 | 1 | 0 | 0 | 0 | 0 | 0 | 43.0 | 0 | Basaloid |
| 50 | Agaimy et al,[16] | 2017 | 28 | 1 | 4a | 0 | 0 | Anterior ethmoidal and right frontal sinuses | Radical resection followed by postoperative chemoradiation | 0 | 1 | 0 | 1 | 1 | 0 | 0 | 0 | 0 | 115.0 | 0 | Basaloid |

| | | | | | | | | | | | | | | | | | | | | | |
|---|---|---|---|---|---|---|---|---|---|---|---|---|---|---|---|---|---|---|---|---|---|
| 51 | Agaimy et al,[16] | 2017 | 52 | 0 | 4a | UK | UK | Nasal cavity and ethmoid sinus | Radical resection | 0 | 0 | 0 | 0 | 1 | 1 | 0 | 0 | 0 | 12.0 | 2 | Basaloid |
| 52 | Agaimy et al,[16] | 2017 | 78 | 1 | 4a | 0 | 0 | Nasal cavity and ethmoidal sinus | Radical resection | 0 | 0 | 0 | 0 | 0 | 0 | 0 | 0 | 0 | 26.0 | 0 | Plasmacytoid/rhabdoid |
| 53 | Agaimy et al,[16] | 2017 | 76 | 1 | 4a | 0 | 1 | All paranasal sinuses | Radical resection followed by postoperative chemoradiation | 0 | 1 | 0 | 1 | 1 | 1 | 0 | 0 | 0 | 4.0 | 2 | Plasmacytoid/rhabdoid |
| 54 | Agaimy et al,[16] | 2017 | 26 | 0 | 2 | UK | UK | Ethmoidal sinus | Radical resection followed by postoperative chemoradiation | 0 | 1 | 0 | 1 | 0 | 0 | 0 | 0 | 0 | 16.0 | 0 | Basaloid with focal clear cell features |
| 55 | Agaimy et al,[16] | 2017 | 87 | 1 | 4a | UK | UK | Nasal cavity and all paranasal sinuses | Radical resection followed by postoperative chemoradiation | 0 | 1 | 0 | 1 | 0 | 0 | 0 | 0 | 0 | 11.0 | 0 | Basaloid |
| 56 | Agaimy et al,[16] | 2017 | 44 | 0 | 4a | 0 | 0 | Ethmoidal sinus | Radical resection followed by postoperative chemoradiation | 0 | 1 | 0 | 1 | 0 | 0 | 0 | 0 | 0 | 23.0 | 0 | Basaloid |
| 57 | Agaimy et al,[16] | 2017 | 45 | 0 | 4a | 0 | 0 | Nasal cavity and left frontal sinus | Radical resection followed by postoperative chemoradiation | 0 | 1 | 0 | 1 | 0 | 0 | 0 | 0 | 0 | 42.0 | 0 | Plasmacytoid/rhabdoid with adenoid features |
| 58 | Agaimy et al,[16] | 2017 | 46 | 1 | 4a | 0 | 0 | Frontal and ethmoidal sinuses | Radical resection followed by | 0 | 1 | 0 | 1 | 0 | 0 | 0 | 0 | 0 | 57.0 | 0 | Basaloid |

| | | | | | | | | | | | | | | | | | | | | | |
|---|---|---|---|---|---|---|---|---|---|---|---|---|---|---|---|---|---|---|---|---|---|
| | | | | | | | | | postoperative radiation therapy | | | | | | | | | | | | |
| 59 | Wasserman et al,[17] | 2017 | 34 | 0 | 4a | 0 | 0 | Maxillary sinus invading into the orbital floor | Preoperative chemoradiation followed by radical resection | 1 | 0 | 0 | 1 | 1 | 0 | 0 | 1 | 1 | 26.0 | 2 | Rhabdoid with comedo necrosis |
| 60 | Wasserman et al,[17] | 2017 | 56 | 1 | 4b | 0 | 0 | Sinonasal mass with intracranial extension and cavernous sinus invasion | Radical concurrent chemoradiation | 0 | 0 | 0 | 1 | 0 | 0 | 0 | 0 | 0 | 12.0 | 0 | Rhabdoid |
| 61 | Dogan et al,[19] | 2017 | 54 | 0 | 4a | 0 | 0 | Nasal cavity and ethmoidal sinus | Radical resection | 0 | 0 | 0 | 0 | 1 | 0 | 0 | 1 | 1 | 39.0 | 2 | - |
| 62 | Dogan et al,[19] | 2017 | 47 | 0 | 4b | 0 | 0 | Ethmoid sinus | Preoperative chemoradiation followed by surgery | 1 | 0 | 0 | 1 | 1 | 1 | 0 | 0 | 0 | 7.0 | 1 | - |
| 63 | Dogan et al,[19] | 2017 | 54 | 0 | 4b | 0 | 0 | Nasal cavity and ethmoidal sinus | Induction chemotherapy followed by surgery | 1 | 0 | 0 | 1 | 1 | 1 | 0 | 1 | 0 | 23.0 | 1 | - |
| 64 | Laco et al,[21] | 2018 | 76 | 0 | 2 | 0 | 0 | Nasal cavity | Radical resection followed by post-operative radiation therapy | 0 | 1 | 0 | 1 | 0 | 0 | 0 | 0 | 1 | 71.0 | 3 | Basaloid and rhabdoid |
| 65 | Laco et al,[21] | 2018 | 27 | 0 | 4a | 0 | 0 | Nasal cavity | Radical resection followed by post-operative chemoradiation | 0 | 1 | 0 | 1 | 1 | 1 | 0 | 0 | 1 | 73.0 | 2 | Basaloid and rhabdoid |

| | | | | | | | | | | | | | | | | | | | | | |
|---|---|---|---|---|---|---|---|---|---|---|---|---|---|---|---|---|---|---|---|---|---|
| 66 | Laco et al,[21] | 2018 | 51 | 0 | 4b | 0 | 0 | Nasal cavity | Radical resection followed by post-operative radiation therapy | 0 | 1 | 0 | 1 | 1 | 1 | 0 | 0 | 1 | 14.0 | 2 | Basaloid and rhabdoid |
| 67 | Laco et al,[21] | 2018 | 54 | 1 | 4a | 0 | 0 | Nasal cavity | Radical resection followed by post-operative radiation therapy | 0 | 1 | 0 | 1 | 1 | 1 | 0 | 0 | 0 | 122.0 | 0 | Basaloid and rhabdoid |
| 68 | Allard et al,[22] | 2018 | 87 | 0 | 4b | UK | UK | Nasal cavity, ethmoidal and maxillary sinuses with intracranial extension | UK | UK | UK | UK | UK | 1 | 1 | 1 | 1 | 1 | 9.0 | 2 | Basaloid |
| 69 | Allard et al,[22] | 2018 | 51 | 1 | 4b | 0 | 1 | Sphenoid sinus with invasion into the temporal fossa | UK | UK | UK | UK | UK | 0 | 0 | 0 | 0 | 1 | 24.0 | 2 | Basaloid |
| 70 | Allard et al,[22] | 2018 | 62 | 0 | 4b | UK | UK | Nasal cavity and ethmoidal sinus | UK | UK | UK | UK | UK | 1 | 1 | 1 | 1 | 1 | 43.0 | 2 | Rhabdoid |
| 71 | Allard et al,[22] | 2018 | 32 | 0 | 4b | UK | UK | Nasal cavity, ethmoidal and frontal sinuses with orbital extension | UK | UK | UK | UK | UK | 0 | 0 | 0 | 0 | 0 | 9.0 | 0 | Basaloid |
| 72 | Allard et al,[22] | 2018 | 75 | 0 | 3 | 1 | 0 | Nasal cavity and ethmoidal sinus | UK | UK | UK | UK | UK | 1 | 0 | 1 | 0 | 0 | 30.0 | 1 | Basaloid |

| | | | | | | | | | | | | | | | | | | | | |
|---|---|---|---|---|---|---|---|---|---|---|---|---|---|---|---|---|---|---|---|---|
| 73 | Allard et al,[22] | 2018 | 64 | 1 | 3 | 0 | 1 | Frontal sinus | UK | UK | UK | UK | UK | 1 | 0 | 0 | 1 | 0 | 36.0 | 1 | Rhabdoid |
| 74 | Kakkar et al,[23] | 2019 | 58 | 0 | 1 | 0 | 0 | Left nasal cavity | Left lateral rhinotomy excision followed by chemoradiation | 0 | 1 | 0 | 1 | 0 | 0 | 0 | 0 | 0 | 8.0 | 0 | Basaloid |
| 75 | Kakkar et al,[23] | 2019 | 23 | 0 | 3 | 0 | 0 | Left nasal cavity, maxillary and ethmoidal sinuses with destruction of bony walls and floor of maxillary sinus | Preoperative chemotherapy followed by radical maxillectomy and postoperative radiation therapy | 1 | 0 | 0 | 1 | 0 | 0 | 0 | 0 | 0 | 17.0 | 0 | Basaloid |
| 76 | Kakkar et al,[23] | 2019 | 20 | 0 | 4b | 0 | 0 | Nasal cavity, right maxillary sinus extending to the infratemporal fossa, skull base, and orbit | No treatment | 0 | 0 | 0 | 0 | 1 | 1 | 0 | 0 | 0 | 2.0 | 2 | Basaloid |
| 77 | McHugh, et al,[24] | 2019 | 53 | 0 | 4b | 0 | 0 | Ethmoidal sinus with invasion into the left medial orbit and frontal cranial fossa | Radical resection with maxillectomy, orbital exenteration and infratemporal fossa dissection | 0 | 0 | 0 | 0 | 1 | 0 | 0 | 1 | 0 | 9.0 | 1 | Basaloid |

| | | | | | | | | | | | | | | | | | | | | | |
|---|---|---|---|---|---|---|---|---|---|---|---|---|---|---|---|---|---|---|---|---|---|
| 78 | Gomez-Acevedo et al,[25] | 2019 | 42 | 1 | 3 | 0 | 0 | Paranasal sinuses with invasion into the trigeminal nerve | Radical resection followed by gammaknife stereotactic radiosurgery | 0 | 1 | 0 | 1 | 1 | 1 | 0 | 0 | 1 | 13.0 | 2 | Undifferentiated carcinoma |
| 79 | Trieu et al,[26] | 2019 | 53 | 0 | 4b | 0 | 0 | Rt posterior ethmoidal sinus with invasion into the orbital apex | Induction chemotherapy TPF followed by radical concurrent chemoradiation with proton therapy | 1 | 0 | 0 | 1 | 1 | 0 | 0 | 1 | 1 | 13.0 | 2 | Basaloid |
| 80 | Vaziri Fard et al,[27] | 2019 | 75 | 0 | 4a | 0 | 0 | Large sinonasal mass with bilateral orbital and skull base involvement | Radical chemoradiation | 0 | 0 | 0 | 1 | 0 | 0 | 0 | 0 | 0 | 7.0 | 1 | Plasmacytoid/rhabdoid |
| 81 | Vaziri Fard et al,[27] | 2019 | 61 | 0 | 4b | 2 | 1 | Large sinonasal mass with orbital and brain extension | Radical chemoradiation | 0 | 0 | 0 | 1 | 0 | 0 | 0 | 0 | 0 | 17.0 | 1 | Plasmacytoid/rhabdoid |
| 82 | Vaziri Fard et al,[27] | 2019 | 61 | 0 | 4b | 0 | 1 | Large sinonasal mass with brain invasion | Surgery followed by chemoradiation | 0 | 1 | 0 | 1 | 0 | 0 | 0 | 0 | 0 | 11.0 | 1 | Plasmacytoid/rhabdoid |
| 83 | Levitan et al,[31] | 2020 | 36 | 0 | 4b | 0 | 0 | Ethmoidal and frontal sinuses with invasion into the right orbit and dura | No treatment | 0 | 0 | 0 | 0 | 0 | 0 | 0 | 0 | 1 | 0.3 | 2 | - |
| 84 | Shanti et al,[32] | 2020 | 63 | 1 | 4b | 0 | 0 | Left maxillary | Induction chemotherapy | 1 | 0 | 0 | 1 | 1 | 1 | 0 | 0 | 0 | 8.0 | 0 | Rhabdoid |

| | | | | | | | | | | | | | | | | | | | | | |
|---|---|---|---|---|---|---|---|---|---|---|---|---|---|---|---|---|---|---|---|---|---|
| | | | | | | | | sinus with invasion of the left orbital floor, inferior rectus and infraorbital nerve | with docetaxel and cisplatin followed by radical concurrent chemoradiation | | | | | | | | | | | | |
| 85 | Chitguppi et al,[33] | 2020 | UD | 0 | 4b | UD | UD | Paranasal sinuses with invasion into the orbital cavity | Radical resection followed by postoperative chemoradiation | 0 | 1 | 0 | 1 | UD | UD | UD | UD | 1 | 1.4 | 2 | - |
| 86 | Chitguppi et al,[33] | 2020 | UD | 0 | 4b | UD | UD | Paranasal sinuses with invasion into the orbital cavity | Radical resection followed by postoperative chemoradiation | 0 | 1 | 0 | 1 | UD | UD | UD | UD | 1 | 2.1 | 2 | - |
| 87 | Chitguppi et al,[33] | 2020 | UD | 1 | 4b | UD | UD | Paranasal sinuses with invasion into the orbital cavity | Induction chemotherapy followed by radical concurrent chemoradiation | 1 | 0 | 0 | 1 | UD | UD | UD | UD | 1 | 56.9 | 0 | - |
| 88 | Chitguppi et al,[33] | 2020 | UD | 1 | 4b | UD | UD | Paranasal sinuses with invasion into the orbital cavity | Induction chemotherapy followed by radical concurrent chemoradiation | 1 | 0 | 0 | 1 | UD | UD | UD | UD | 1 | 81.4 | 0 | - |
| 89 | Chitguppi et al,[33] | 2020 | UD | 0 | 4b | UD | UD | Paranasal sinuses with invasion into the orbital cavity | Induction chemotherapy followed by radical concurrent chemoradiation | 1 | 0 | 0 | 1 | UD | UD | UD | UD | 0 | 9.7 | 2 | - |

| | | | | | | | | | | | | | | | | | | | | | |
|---|---|---|---|---|---|---|---|---|---|---|---|---|---|---|---|---|---|---|---|---|---|
| 90 | Chitguppi et al,[33] | 2020 | UD | 0 | 4b | UD | UD | Paranasal sinuses with invasion into the orbital cavity | Induction chemotherapy followed by radical concurrent chemoradiation | 1 | 0 | 0 | 1 | UD | UD | UD | UD | 0 | 25.1 | 0 | - |
| 91 | Libera et al,[36] | 2021 | 70 | 0 | 4b | 0 | 0 | Maxillary sinus | UK | UK | UK | UK | UK | UK | UK | UK | UK | 1 | 4.0 | UD | - |
| 92 | Libera et al,[36] | 2021 | 66 | 0 | 4b | 0 | 0 | Ethmoidal sinus | UK | UK | UK | UK | UK | UK | UK | UK | UK | 1 | 11.0 | UD | - |
| 93 | Libera et al,[36] | 2021 | 40 | 0 | 4b | 0 | 0 | Maxillary sinus | UK | UK | UK | UK | UK | UK | UK | UK | UK | 1 | 14.0 | UD | - |
| 94 | Libera et al,[36] | 2021 | 72 | 0 | 4b | 0 | 0 | Frontal sinus | UK | UK | UK | UK | UK | UK | UK | UK | UK | 1 | 19.0 | UD | - |
| 95 | Libera et al,[36] | 2021 | 48 | 0 | 4b | 0 | 0 | Maxillary sinus | UK | UK | UK | UK | UK | UK | UK | UK | UK | 1 | 26.0 | UD | - |
| 96 | Libera et al,[36] | 2021 | 42 | 1 | 4b | 0 | 0 | Frontal sinus | UK | UK | UK | UK | UK | UK | UK | UK | UK | 1 | 41.0 | UD | - |
| 97 | Libera et al,[36] | 2021 | 43 | 0 | 2 | 0 | 0 | Ethmoidal sinus | UK | UK | UK | UK | UK | UK | UK | UK | UK | 1 | 42.0 | UD | - |
| 98 | Libera et al,[36] | 2021 | 76 | 0 | 4b | 0 | 0 | Ethmoidal sinus | UK | UK | UK | UK | UK | UK | UK | UK | UK | 0 | 91.0 | UD | - |
| 99 | Libera et al,[36] | 2021 | 46 | 1 | 4a | 0 | 0 | Ethmoidal sinus | UK | UK | UK | UK | UK | UK | UK | UK | UK | 0 | 149.0 | UD | - |
| 100 | Li et al,[37] | 2021 | 55 | 0 | 4a | 0 | 0 | Right nasal cavity invading into maxillary and sphenoidal sinuses and right orbital wall | Radical resection followed by postoperative radiation therapy | 0 | 1 | 0 | 1 | 0 | 0 | 0 | 0 | 0 | 6.0 | 0 | Yolk sac differentiation |
| 101 | Ayyanar, et al,[39] | 2021 | 61 | 0 | 2 | 0 | 0 | Frontal and ethmoidal sinuses | Radical resection followed by postoperative | 0 | 1 | 0 | 1 | 1 | 1 | 0 | 0 | 1 | 24.0 | 2 | Small cells |

| | | | | | | | | | | | | | | | | | | | | | |
|---|---|---|---|---|---|---|---|---|---|---|---|---|---|---|---|---|---|---|---|---|---|
| | | | | | | | | | chemoradiation | | | | | | | | | | | | |
| 102 | Ayyanar, et al,[39] | 2021 | 35 | 1 | 4b | 0 | 0 | Nasal cavity, right ethmoidal sinus with intracranial extension | Anterior cranio-facial resection of right sided nasal mass and right orbital exenteration followed by chemoradiation | 0 | 1 | 0 | 1 | 1 | 1 | 0 | 0 | 0 | 5.0 | 1 | Pseudoalveolar pattern, pseudoglandular pattern, empty vacuoles |
| 103 | Ayyanar, et al,[39] | 2021 | 35 | 1 | 3 | 0 | 0 | Left nasal cavity with extension into sphenoidal and posterior ethmoid sinuses | Radical resection followed by postoperative chemoradiation | 0 | 1 | 0 | 1 | 0 | 0 | 0 | 0 | 0 | 20.0 | 0 | Basaloid |
| 104 | Hazir et al,[40] | 2021 | 55 | 0 | 4a | 0 | 0 | Left maxillary sinus invading into the left orbit | Preoperative chemotherapy followed by surgery with orbital exenteration and postoperative chemoradiation | 1 | 1 | 1 | 1 | 1 | 1 | 0 | 1 | 1 | 12.0 | 2 | Basaloid and squamoid |
| 105 | Su et al,[41] | 2021 | 54 | 1 | 4b | 0 | 0 | Left nasal cavity with invasion into the ethmoidal, maxillary sinuses and orbital cavity and | Left lateral rhinotomy, left partial maxillectomy, and pansinusectomy | 0 | 0 | 0 | 0 | 0 | 0 | 0 | 0 | 1 | 11.0 | 0 | Oncocytoid and rhabdoid |

| | | | | | | | | | | | | | | | | | | | | | |
|---|---|---|---|---|---|---|---|---|---|---|---|---|---|---|---|---|---|---|---|---|---|
| | | | | | | | | right medial orbital wall | | | | | | | | | | | | | |
| 106 | Wang et al,[42] | 2022 | 47 | 1 | 4a | 0 | 0 | Left ethmoidal and sphenoid sinuses | Endoscopic resection followed by postoperative radiotherapy | 0 | 1 | 0 | 1 | 1 | 0 | 0 | 1 | 1 | 32.0 | 2 | - |
| 107 | Wang et al,[42] | 2022 | 54 | 1 | 4a | 0 | 0 | Right ethmoidal sinus | Endoscopic surgery followed by postoperative radiotherapy | 0 | 1 | 0 | 1 | 1 | 0 | 0 | 1 | 1 | 7.0 | 2 | - |
| 108 | Wang et al,[42] | 2022 | 59 | 1 | 4a | 0 | 0 | Bilateral ethmoidal sinuses | Induction chemotherapy followed by radical chemoradiation | 1 | 0 | 0 | 1 | 1 | 0 | 0 | 1 | 1 | 9.0 | 2 | - |
| 109 | Wang et al,[42] | 2022 | 38 | 0 | 4b | 2 | 0 | Bilateral ethmoidal sinuses | Preoperative chemotherapy followed by resection and postoperative chemoradiation | 1 | 1 | 1 | 1 | 1 | 1 | 1 | 1 | 1 | 31.0 | 2 | - |
| 110 | Wang et al,[42] | 2022 | 64 | 0 | 4a | 0 | 0 | Left maxillary sinus | Radical resection followed by postoperative chemoradiation and chemotherapy | 0 | 1 | 0 | 1 | 1 | 1 | 0 | 11 | 1 | 14.0 | 2 | - |
| 111 | Wang et al,[42] | 2022 | 59 | 0 | 4a | 0 | 0 | Right ethmoidal sinus | Radical endoscopic resection | 0 | 0 | 0 | 0 | 0 | 0 | 0 | 0 | 0 | 18.0 | 0 | - |
| 112 | Wang et al,[42] | 2022 | 38 | 0 | 3 | 0 | 0 | Left ethmoidal and maxillary sinuses | Radical endoscopic resection | 0 | 0 | 0 | 0 | 0 | 0 | 0 | 0 | 0 | 28.0 | 0 | - |

| | | | | | | | | | | | | | | | | | | | | | |
|---|---|---|---|---|---|---|---|---|---|---|---|---|---|---|---|---|---|---|---|---|---|
| 113 | Wang et al,[42] | 2022 | 78 | 0 | 4a | 0 | 0 | Right maxillary, ethmoidal and sphenoid sinuses | Radical endoscopic surgery and open surgery | 0 | 0 | 0 | 1 | 1 | 1 | 0 | 0 | 0 | 56.0 | 1 | - |
| 114 | Wang et al,[42] | 2022 | 43 | 1 | 4a | 0 | 0 | Right frontal and ethmoid sinuses | Radical endoscopic resection followed by postoperative chemoradiation | 0 | 1 | 0 | 1 | 0 | 0 | 0 | 0 | 0 | 3.0 | 0 | - |
| 115 | Wang et al,[42] | 2022 | 65 | 1 | 2 | 0 | 0 | Right ethmoidal sinus | Radical endoscopic resection followed by postoperative chemotherapy | 0 | 1 | 0 | 1 | 0 | 0 | 0 | 0 | 0 | 18.0 | 0 | - |
| 116 | Wang et al,[42] | 2022 | 53 | 1 | 3 | UD | UD | Bilateral ethmoidal sinuses | Radical endoscopic resection followed by postoperative radiotherapy, open surgery and chemotherapy | 0 | 1 | 0 | 1 | 1 | 1 | 0 | 0 | 0 | 27.0 | 1 | - |
| 117 | Wang et al,[42] | 2022 | 25 | 0 | 4a | 0 | 0 | Right frontal and ethmoidal sinus | Preoperative chemotherapy followed by open surgery and postoperative chemoradiation | 1 | 1 | 1 | 1 | 1 | 1 | 0 | 1 | 0 | 35.0 | 1 | - |
| 118 | Wang et al,[42] | 2022 | 53 | 0 | 4a | 0 | 0 | Bilateral ethmoidal and frontal sinuses | Preoperative chemotherapy followed by resection | 1 | 0 | 0 | 1 | 0 | 0 | 0 | 0 | 0 | 30.0 | 0 | - |

| | | | | | | | | | | | | | | | | | | | | | |
|---|---|---|---|---|---|---|---|---|---|---|---|---|---|---|---|---|---|---|---|---|---|
| 119 | Wang et al,[42] | 2022 | 42 | 0 | 4a | 2 | 1 | Bilateral ethmoidal and sphenoid sinuses, right frontal and maxillary sinuses | Palliative chemotherapy | 0 | 0 | 0 | 0 | 1 | 0 | 1 | 0 | 0 | 21.0 | 1 | - |
| 120 | Wang et al,[42] | 2022 | 41 | 1 | 4a | 0 | 0 | Left ethmoidal sinus | Radiation therapy followed by surgery | 1 | 0 | 0 | 1 | 0 | 0 | 0 | 0 | 0 | 16.0 | 0 | - |
| 121 | HKU cohort (current study) | 2022 | 45 | 0 | 4b | 0 | 0 | Nasal cavity with invasion into the maxillary sinus, ethmoid sinus, orbital floor and intracranial cavity | Preoperative chemotherapy TPF × 3 cycles followed by craniofacial resection and orbital exenteration and postoperative chemoradiation | 1 | 1 | 1 | 1 | 0 | 0 | 0 | 0 | 1 | 8.9 | 0 | Undifferentiated carcinoma |
| 122 | HKU cohort (current study) | 2022 | 51 | 0 | 4b | 0 | 0 | Nasal cavity with invasion into the maxillary sinus, ethmoid sinus and orbital floor | Preoperative chemotherapy PF x 3 cycles followed by craniofacial resection and postoperative chemoradiation with carboplatin | 1 | 1 | 1 | 1 | 1 | 0 | 0 | 1 | 1 | 10.7 | 2 | Undifferentiated carcinoma |
| 123 | HKU cohort (current study) | 2022 | 58 | 1 | 4a | 0 | 0 | Nasal cavity with invasion into the maxillary sinus and | Craniofacial resection followed by chemoradiation with proton therapy | 0 | 1 | 0 | 1 | 1 | 1 | 0 | 0 | 0 | 56.4 | 0 | High-grade non-intestinal adenocarcinoma |

| | | | | | | | | | | | | | | | | | | | | | |
|---|---|---|---|---|---|---|---|---|---|---|---|---|---|---|---|---|---|---|---|---|---|
| | | | | | | | ethmoidal sinus | | | | | | | | | | | | | | |
| 124 | HKU cohort (current study) | 2022 | 42 | 0 | 4b | 0 | 0 | Nasal cavity with invasion into the maxillary sinus and right medial orbital floor | Induction TPF x 3 cycles followed by concurrent chemoradiation | 1 | 0 | 0 | 1 | 0 | 0 | 0 | 0 | 0 | 45.7 | 0 | Basaloid |
| 125 | HKU cohort (current study) | 2022 | 30 | 1 | 3 | 0 | 0 | Nasal cavity invading into maxillary sinus | Preoperative chemotherapy with TPX × 3 cycles followed endoscopic maxillectomy and postoperative chemoradiation | 1 | 1 | 1 | 1 | 0 | 0 | 0 | 0 | 0 | 21.4 | 0 | Basaloid |
| 126 | HKU cohort (current study) | 2022 | 36 | 1 | 4b | 0 | 0 | Nasal cavity with invasion into maxillary sinus, ethmoid sinus and bilateral anterior frontal fossae | Radical resection followed by postoperative proton therapy | 0 | 1 | 1 | 1 | 1 | 1 | 0 | 0 | 0 | 60.1 | 1 | Squamous |
| 127 | HKU cohort (current study) | 2022 | 63 | 0 | 4b | 0 | 0 | Nasal cavity with invasion into the maxillary sinus, ethmoid sinus, | Preoperative chemotherapy TPF × 3 cycles followed by radical chemoradiation | 1 | 0 | 0 | 1 | 0 | 0 | 0 | 0 | 0 | 36.9 | 0 | Undifferentiated carcinoma |

| | | | | | | | | | | | | | | | | | | | | | |
|---|---|---|---|---|---|---|---|---|---|---|---|---|---|---|---|---|---|---|---|---|---|
| | | | | | | | | orbital floor and intracranial cavity | | | | | | | | | | | | | |
| 128 | HKU cohort (current study) | 2022 | 53 | 0 | 4b | 0 | 0 | Nasal cavity with invasion into the maxillary sinus and ethmoidal sinus | Preoperative chemotherapy TPF × 3 cycles followed by radical craniofacial resection and postoperative chemoradiation | 1 | 1 | 1 | 1 | 0 | 0 | 0 | 0 | 0 | 24.7 | 0 | Undifferentiated carcinoma |

*References are in the same order as those cited in the manuscript.

***PF* cisplatin and 5-fluorouracil, *TPF* docetaxel, cisplatin, and 5-fluorouracil, *TPX* docetaxel, cisplatin, and capecitabine, *UD* undetermined, *UK* UnknownReferences**

1. Agaimy, A.; Koch, M.; Lell, M.; Semrau, S.; Dudek, W.; Wachter, D.L.; Knöll, A.; Iro, H.; Haller, F.; Hartmann, A. SMARCB1(INI1)-deficient sinonasal basaloid carcinoma: a novel member of the expanding family of SMARCB1-deficient neoplasms. *Am. J. Surg. Pathol.* **2014**, *38*, 1274–1281.

2. Bishop, J.A.; Antonescu, C.R.; Westra, W.H. SMARCB1 (INI-1)-deficient carcinomas of the sinonasal tract. *Am. J. Surg. Pathol.* **2014**, *38*, 1282–1289.

3. Judkins, A.R. Immunohistochemistry of INI1 expression: a new tool for old challenges in CNS and soft tissue pathology. *Adv. Anat. Pathol.* **2007**, *14*, 335–339.

4. El-Naggar, A.K.; Chan, J.K.C.; Grandis, J.R.; Takata, T.; Slootweg, P.J.; editors. WHO classification of head and neck tumours. Lyon, France: IARC, **2017**.

5. Morand, G.B.; Anderegg, N.; Vital, D.; Ikenberg, K.; Huber, G.F.; Soyka, M.B.; Egger, M.; Holzmann, D. Outcome by treatment modality in sinonasal undifferentiated carcinoma (SNUC): A case-series, systematic review and meta-analysis. *Oral Oncol.* **2017**, *75*, 28–34.

6. Reiersen, D.A.; Pahilan, M,E; Devaiah, A.K. Meta-analysis of treatment outcomes for sinonasal undifferentiated carcinoma. *Otolaryngol. Head Neck Surg.* **2012**, *147*, 7–14.

7. Kuo, P.; Manes, R.P.; Schwam, Z.G.; Judson, B.J. Survival outcomes for combined modality therapy for sinonasal undifferentiated carcinoma. *Otolaryngol. Head Neck Surg.* **2017**, *156*, 132–136.

8. Bhasker, S.; Mallick, S.; Benson, R.; Bhanuprasad, V.; Sharma, A.; Thakar, A. A multimodality approach to sinonasal undifferentiated carcinoma: A single institute experience. *J. Laryngol. Otol.* **2017**; *131*, 19–25.

9. Amit, M.; Abdelmeguid, A.S.; Watcherporn, T.; Takahashi, H.; Tam, S.; Bell, D.; Ferrarotto, R.; Glisson, B.; Kupferman, M.E.; et al. Induction chemotherapy response as a guide for treatment optimization in sinonasal undifferentiated carcinoma. *J. Clin. Oncol.* **2019**, *37*, 504–512.

10. Chemotherapy Before Surgery and Radiation Therapy or Surgery and Radiation Therapy Alone in Treating Patients With Nasal and Paranasal Sinus Cancer That Can Be Removed by Surgery. Available online: https://clinicaltrials.gov/ct2/show/NCT03493425 (accessed 15 May 2022).
